# Progress or Pathology? Differential Diagnosis and Intervention Criteria for Meditation-Related Challenges: Perspectives From Buddhist Meditation Teachers and Practitioners

**DOI:** 10.3389/fpsyg.2020.01905

**Published:** 2020-07-29

**Authors:** Jared R. Lindahl, David J. Cooper, Nathan E. Fisher, Laurence J. Kirmayer, Willoughby B. Britton

**Affiliations:** ^1^Department of Religious Studies, Brown University, Providence, RI, United States; ^2^Department of Psychiatry and Human Behavior, Brown University, Providence, RI, United States; ^3^Department of Religious Studies, University of California, Santa Barbara, Santa Barbara, CA, United States; ^4^Division of Social and Transcultural Psychiatry, McGill University, Montreal, QC, Canada

**Keywords:** religious experiences, adverse experiences, meditation, Buddhism, mental health, psychiatric diagnosis

## Abstract

Studies in the psychology and phenomenology of religious experience have long acknowledged similarities with various forms of psychopathology. Consequently, it has been important for religious practitioners and mental health professionals to establish criteria by which religious, spiritual, or mystical experiences can be differentiated from psychopathological experiences. Many previous attempts at differential diagnosis have been based on limited textual accounts of mystical experience or on outdated theoretical studies of mysticism. In contrast, this study presents qualitative data from contemporary Buddhist meditation practitioners and teachers to identify salient features that can be used to guide differential diagnosis. The use of certain existing criteria is complicated by Buddhist worldviews that some difficult or distressing experiences may be expected as a part of progress on the contemplative path. This paper argues that it is important to expand the framework for assessment in both scholarly and clinical contexts to include not only criteria for determining normative fit with religious experience or with psychopathology, but also for determining need for intervention, whether religious or clinical. Qualitative data from Buddhist communities shows that there is a wider range of experiences that are evaluated as potentially warranting intervention than has previously been discussed. Decision making around these experiences often takes into account contextual factors when determining appraisals or need for intervention. This is in line with person-centered approaches in mental health care that emphasize the importance of considering the interpersonal and cultural dynamics that inevitably constitute the context in which experiences are evaluated and rendered meaningful.

## Introduction

An important question in the study of religious experiences is when and how to differentiate religious experiences from psychopathology. This need for “differential diagnosis” arises in the contexts of religious practice when some individuals have challenging or adverse experiences and also in clinical settings where individuals seek help for persistent distress. This paper presents data from the *Varieties of Contemplative Experience* (VCE) project (Lindahl et al., 2017), a mixed-method study of meditation-related challenges among Western Buddhists, to demonstrate how differential diagnosis decisions are made by meditators and meditation teachers, some of whom were also clinicians. Investigating Buddhist meditation on this topic is revealing, as there are many challenging experiences that are often considered “part of the path” or normative stages of contemplative development by practitioners and teachers in these communities. However, contrary to the dominant narrative extolling the positive impacts of meditation, there are also challenging experiences that are in some cases considered unwanted adverse effects (Lindahl et al., 2019). The purpose of this paper is to identify the specific criteria that contemporary Buddhist meditation teachers and practitioners in the West use in order to differentiate expected, intended, or religiously significant experiences from potentially concerning forms of psychopathology. First, we will concisely summarize prior attempts to establish differential diagnosis of religious experience and psychopathology based upon either primary or secondary features of the experience. Then, we will explain how we assessed the differential diagnosis of meditation-related challenges in the real-world context of Buddhist meditation communities by applying criteria from existing research toward an analysis of the VCE data set. Drawing extensively from qualitative data from both meditation teachers and meditation practitioners, we will demonstrate that in many instances, the concern was not about identifying a given experience as either “religious” or “psychopathological,” but with determining whether a particular experience, regardless of appraisal, warranted additional support or intervention. We will also show how that there is a wider range of experiences that are evaluated as potentially warranting intervention than has previously been discussed. Finally, we will discuss the implications of our findings both for the clinical assessment of meditation-related challenges and for Buddhist meditation communities.

### Previous Research on the Differential Diagnosis of Religious Experiences and Psychopathology

Starting in the 1980s, seminal work by Grof and Grof (2017, 1989) identified ten “varieties of spiritual emergency.”^1^ This approach was positioned as a challenge to the culturally insensitive and narrow definitions of mental health in mainstream psychology that ignored or pathologized “non-ordinary” states of consciousness. The Grofs and others in the transpersonal psychology movement were adamant that these experiences reflected access to legitimate realities and sacred knowledge that would result in healing, but only if they were properly supported, i.e., were allowed to follow their natural course and were not suppressed by medication (Grof and Grof, 1989, p. 7). The concern then was with normative judgements and prescriptions for clinical responses that would facilitate rather than interfere with positive spiritual growth.

The Grofs’ descriptions of these experiences either relied upon specific associated phenomenology (e.g., the awakening of *kuṇḍalinī* is indicated by “intense sensations of energy and heat streaming up the spine” and by “visions of brilliant light”) or conflated phenomenology with appraisals of outcomes (e.g., shamanic crises “often culminate in experiences of death and dismemberment followed by rebirth and ascent to celestial regions”) (pp. 14−15). In this approach, most of the emphasis is placed on the specific phenomenological features of spiritual emergencies or on their positive impacts on the person’s spiritual life, although additional scholarship has identified other distinguishing features such as an absence of comorbidities or the ability to maintain a critical attitude toward the experience (Lukoff et al., 1998; Johnson and Friedman, 2008; Viggiano and Krippner, 2010; Grof and Grof, 2017).

The spiritual emergency movement also informed attempts to revise the *Diagnostic and Statistical Manual of Mental Disorders* (*DSM*) of the American Psychiatric Association. The *DSM-IV* included a number of additions that were intended to improve cultural considerations, including a glossary of culture-bound syndromes, an outline of cultural formulation, V-codes describing situational problems or predicaments, and notes on cultural qualifiers for specific disorders. Turner et al. (1995) proposed a new category for this edition called “Religious or Spiritual Problem,” which could be distressing and impairing enough to warrant clinical attention, but would be classified as “non-pathological” by way of using a V-code (American Psychiatric Association [APA], 1994, V62.89, p. 685). Although the V-code for “Religious or Spiritual Problem” is sometimes used to indicate the need for differential diagnosis between religious experience and psychopathology (Prusak, 2016), the description of this category is geared more toward the loss or questioning of religious values than toward evaluating unusual experiences.

Other sections of the *DSM-IV* and *DSM-5* suggest criteria for differential diagnosis by introducing cultural caveats to specific diagnoses. For instance, in the section on brief psychotic disorder, the cultural considerations note states that “in some religious ceremonies, an individual may report hearing voices, but these do not generally persist and are not perceived as abnormal by most members of the individual’s community” (American Psychiatric Association [APA], 2013, §298.8, p. 95). In the section on depersonalization disorder, the cultural comment is specific to meditation:

Voluntarily induced experiences of depersonalization/derealization can be a part of meditative practices that are prevalent in many religions and cultures and should not be diagnosed as a disorder. However, there are individuals who initially induce these states intentionally but over time lose control over them and may develop a fear and aversion for related practices (American Psychiatric Association [APA], 2013, § 300.6, p. 304).

Thus, key criteria that serve as possible exceptions to the cultural caveats include prolonged duration, loss of control, and distress.

Over the years, other researchers have proposed ways in which mystical experiences can be differentiated from psychosis. In line with some of the previous arguments about spiritual emergencies, Brett (2002) contends that although mystical experiences and psychosis “involve an identical disintegration of the mundane worldview,” the former can be “passed through and reintegrated because an appropriate perspective is taken toward the process,” entailing “a suspension of identificatory processes” (p. 336). Parnas and Henriksen (2016) note that mystical experience and schizophrenia share some phenomenological features, specifically a “profoundly diminished sense of embodied, vital self-presence” (p. 78). The primary difference, they suggest, is that whereas mystical experiences are “willingly strived for and at least partially controlled, the self-disorders are typically uncontrollable and involuntary, causing immense suffering” (p. 78). Similar to Brett (2002) and Heriot-Maitland (2008) suggests that mystics will “almost certainly have a *context* to provide meaning for the experience, thus allowing the development of a *structured* appraisal,” whereas the absence of a context for other individuals will leave the appraisal “dangerously open to suggestion,” leading to various delusions (pp. 317−318).

One issue with all three of these articles is the imbalance in their sources of information about psychosis compared to mystical experience. For instance, while Parnas and Henriksen (2016) draw extensively on current empirical research on the phenomenology of psychosis, its mechanisms, and outcomes in contemporary psychiatric patients, their portrait of mystical experience is almost exclusively based on the theories of older textual sources such as W.T. Stace’s *Mysticism and Philosophy*, which problematically delimits mystical experiences to those with positive valence (Taves, 2020). Similarly, Brett (2002) develops her account from a limited number of secondary sources on Buddhist and Hindu traditions in a way that precludes a close comparison of phenomenology (McGhee, 2002). No empirical research on mystics is cited that could be used to support the claims that the phenomenological distinctions are in fact associated with the differences in positive outcomes. As pointed out by Menezes and Moreira-Almeida (2009), “there is a scarcity of empirical studies that prospectively test the differentiating criteria” (p. 88). It is not possible to establish a valid and thorough differentiation of mysticism and psychosis when the phenomenology of psychopathology is based on empirical studies and the phenomenology of mystical experiences is based on religious ideals.

Other approaches to differential diagnosis have emphasized associated features such as duration, distress, functional impairment, or cultural compatibility (Jackson and Fulford, 1997; Littlewood, 1997; Dein, 2012). Researchers adopting these approaches also tend to acknowledge the importance of social and cultural factors in the appraisal of experiences (Rashed, 2010; Dein, 2012; Taylor and Murray, 2012). Jackson and Fulford (1997), for example, identify eight criteria, one of which is based on primary phenomenological features (visual versus auditory hallucinations), while the other seven are based on secondary features that indicate whether the experience is: (1) “acceptable to sub-cultural group” or has “bizarre content”; (2) viewed “as mental contents” or “as veridical perceptions”; (3) accompanied by “possibility of doubt” with “insight present” or is lacking insight; (4) of “brief” or “extended duration”; (5) “volitional” or “involitional”; (6) “other oriented” or “self-oriented”; and (7) “life enhancing” or leads to “deterioration.” Ultimately, Jackson and Fulford (1997) emphasize that “in the case of pathological psychotic phenomena, there is a radical failure of action. In the case of spiritual psychotic phenomena, action is radically enhanced” (p. 55). However, their reliance on the action criterion has also been challenged (Marzanski and Bratton, 2002; Rashed, 2010).

Vieten and Scammell (2015) follow the Grofs by including the classic list of ten spiritual emergencies. In addition, and in line with more recent research, they include nine secondary criteria to be considered in the process of differentiating spiritual experiences from psychopathology: (1) potential medical issues; (2) level of functioning; (3) finding meaning in the experience; (4) coherence; (5) capacity for self-reflection; (6) circumstances of onset; (7) duration; (8) knowledge about the experience; and (9) social context (pp. 66−73).

Finally, in their reviews of the literature, Menezes and Moreira-Almeida (2009, 2010) do not include primary phenomenological characteristics at all; instead, they offer nine secondary criteria that they contend can be used to establish differential diagnosis between psychotic and spiritual experiences. Spiritual but not psychotic experiences are characterized by: (1) lack of suffering; (2) lack of functional impairment; (3) short duration and sporadic occurrence; (4) a critical attitude exists regarding the objective reality of the experience; (5) compatibility with the patient’s cultural background; (6) absence of psychiatric comorbidity; (7) control over the experience; (8) the experience promotes personal growth over time; and (9) the experience is directed toward others (2009, pp. 88−89). Here, as elsewhere in this literature, it is unclear which criteria are necessary or sufficient to determine that an experience is religious or pathological. For a summary of which criteria are present or absent in previous research on differential diagnosis, see Table 1.

**TABLE 1:** Criteria for differentiating psychopathologies from religious, spiritual or mystical experiences considered across different studies.

	Spiritual Emergency^a^	Mysticism and Psychosis^b^	DSM-5^c^	Jackson and Fulford (1997)	Vieten and Scammell (2015)	Menezes and Moreira-Almeida (2009, 2010)	VCE Study
Phenomenological Qualities	+	+	+	+	+	−	+
Distress	x	−	+	−	×	+	+
Control	−	+	+	+	−	+	+
Duration	+	+	+	+	+	+	+
Impact	+	+	−	+	+	+	+
Functional Impairment	×/+	+	+	+	+	+	+
Health History or Condition	+	−	−	−	+	+	+
Critical Attitude	+	−	+	+	+	+	+
Circumstances of Onset	x	+	+	−	+	−	+
Cultural Compatibility	+	−	+	+	+	+	+
Teachers’ Skills or Resources	−	−	−	−	−	−	+

*+, addressed in the source(s) as a possible criterion; −, not discussed in the source(s); x, addressed in the source(s), but rejected as a possible criterion. ^*a*^Grof and Grof (1989), Grof and Grof (2017), Lukoff et al. (1998), Johnson and Friedman (2008). ^*b*^Brett (2002), Heriot-Maitland (2008), Parnas and Henriksen (2016). ^*c*^Considered in particular were the “culture-related diagnostic issues” associated with Brief Psychotic Disorder (§298.8), Schizophrenia (§295.90), Dissociative Identity Disorder (§300.14), Depersonalization/Derealization Disorder (§300.6) and Religious or Spiritual Problem (§V62.89). In the DSM more generally, “differential diagnosis” functions as means of differentiating between types of psychopathology rather than between psychopathology and religious experience.*

## Materials and Methods

### The Varieties of Contemplative Experience Project

The *Varieties of Contemplative Experience* (VCE) is a mixed-methods study of Western meditation practitioners (*n* = 60) and meditation experts (*n* = 32) who could report on meditation-related challenges as well as on how they are interpreted and managed. The research methodology for this project was approved by the Brown University Institutional Review Board. As reported in Lindahl et al. (2017), purposive sampling was used to recruit male (57%) and female meditators practicing in Theravāda, Zen, and Tibetan Buddhist traditions, who were dominantly white and from the United States. Practitioners in these traditions had a wide range of expertise prior to the onset of meditation-related challenges, most (72%) but not all of which occurred in the context of intensive retreat. A minority of practitioners reported a prior psychiatric history (32%) or a trauma history (43%) The primary questions asked during the semi-structured interviews with meditation practitioners concerned challenging, difficult, or distressing experiences associated with meditation, the interpretation of those experiences, and how they were managed. Interviews with experts—meditation teachers both with and without clinical training—included similar questions but pertained to the identification and management of experiences that they had seen in their students. For a comprehensive description of the VCE study’s methods, practitioner demographics, and overall results, see Lindahl et al. (2017). For analysis of select experiences, see Lindahl et al. (2014), Lindahl (2017), Lindahl and Britton (2019). Additional analyses of meditation-related challenges and influencing factors are forthcoming. The purpose of the present analysis is to identify the criteria practitioners and teachers used for making differential diagnosis or for determining need for intervention.

The VCE study documented 59 discrete meditation-related challenges and 26 influencing factors that could affect the nature, duration, or trajectory of those challenges. Reports of meditation experiences were organized into seven higher-order domains: cognitive; perceptual; affective; somatic; conative; sense of self; and social. Some important meditation-related challenges that will be discussed below include: delusional, irrational, or paranormal beliefs; change in executive functioning; hallucinations, visions, or illusions; somatosensory changes; fear, anxiety, panic, or paranoia; re-experiencing of traumatic memories; somatic energy; sleep changes; change in sense of embodiment; and social impairment. Influencing factors identified potentially significant variables associated with the meditation practitioner, their meditation practice, their social relationships, and their health behaviors.

Although the VCE study interview protocol did not include a question on criteria for differential diagnosis or determining need for intervention, when appropriate interviewers sometimes asked follow-up questions such as: “What was she saying that made you think ‘this is mental illness; this is not a spiritual experience that she’s having’?” The topic arose explicitly or implicitly in all but two practitioner interviews (avg. coverage = 14.9%) and all but one expert interview (avg. coverage = 18%)^2^.

### Differential Diagnosis Coding Structure and Approach

A coding scheme for discussions related to differential diagnosis was developed based on a combination of theory-driven and data-driven categories. Because their review papers offered a clear taxonomy of differential diagnosis criteria, we started with theory-driven coding that operationalized the nine criteria from Menezes and Moreira-Almeida (2009, 2010) into the following eight categories: (1) *Distress*; (2) *Functional Impairment*; (3) *Duration*; (4) *Critical Attitude*; (5) *Cultural Compatibility*; (6) *Health History or Condition*; (7) *Control*; and (8) *Impact*. For *Impact*, we combined their criteria of “promotes personal growth over time” and “experience is directed toward others.” In our view, the key distinguishing feature of *Impact* is that it entails a diachronic or retrospective assessment of derivative effects of the experience on the practitioner, whether positive or negative (cf. Jackson and Fulford, 1997). During the process of applying this coding structure, we also allowed for data-driven coding to emerge from the interviews. Two other criteria—(9) *Phenomenological Qualities*, and (10) *Circumstances of Onset*—replicated previous research on differential diagnosis. Finally, one novel category not previously discussed in the literature on differential diagnosis also emerged: (11) *Teachers’ Skills or Resources*. For a complete description of these eleven criteria, see Table 2, and for a comparison of these criteria to previous research, see Table 1.

**TABLE 2:** Descriptions and examples of criteria used for differential diagnosis in the *Varieties of Contemplative Experience* study.

Criterion	Description	Example
Circumstances of Onset	The appraisal of the nature of an experience or determining need for intervention is based upon the context in which it occurred, such as during a meditation retreat or during meditation practice. Also includes references to the impact of environmental or life stressors on meditation practice as a factor determining appraisal or need for intervention.	*By and large we view pretty much everything that comes up on retreat as being part of retreat.* […] *We frame the entirety of the practice as purification of mind—the idea being that whatever is arising is part of what’s wanting to be purified, what’s coming up organically.*

Control	The meditator’s degree of control (or lack thereof) over a challenging experience is identified as a factor in appraising the nature of an experience or in determining need for intervention. Includes the ability (or inability) to modulate or change an experience through altering or stopping meditation practice, through changing one’s environment, or through other behaviors.	*I have all kinds of questions. Like: Can nerves be too open? Can senses be too open? I don’t know. I’ve meditated my whole life, and I’ve meditated full-time for half my adult life. And I’ve never had experiences I can’t come back from. Never. But I can’t get out of this.*

Critical Attitude	The presence or absence of intact reality testing or critical attitude determines how an experience is appraised or need for intervention. Also includes a practitioner’s ability (or lack thereof) to apply different kinds of meditative awareness to gain insight into the nature of their experience.	*Anything that sounds certainly out of touch with reality, delusional, et cetera, we would ask a little bit more about the student to explain* […] *And try to get a sense about, you know: “Do we just have kind of a belief system here, that’s not psychotic, that’s a little weird? Or is this something that this person’s heading into trouble?”*

Cultural Compatibility	The appraisal of the nature of an experience or determining need for intervention is based upon the compatibility or incompatibility of an experience with the practitioner’s or teacher’s cultural background, such as the beliefs, values, and/or behaviors of one’s meditation community, practice tradition, or broader cultural identity.	*The way it was for me is not the way it would ever be for another person, and yet I think it fits in with all these stories of these sort of sudden enlightenment experiences, though, you know, we don’t like to use that word very much. I guess we would call it an enlightenment experience rather than enlightenment. It’s a little easier to deal with. But I feel like it fits into that framework.*

Distress	The presence or absence of distress— including statements about having or not having the capacity to hold an experience or about being overwhelmed—determines how an experience is appraised or need for intervention. Also includes mention of specific indicators of distress, such as dysregulated arousal, especially if they are secondary responses to other phenomenology.	*Of course meditation is going to rock your world a little bit. And to help people understand that while it might be unsettling, it doesn’t mean that anything is wrong. And it’s not easy at times to distinguish between the two. Sometimes it requires a great deal of discernment to find out: is this person getting agitated because they’re moving in one direction or the other? Because I don’t want to normalize something if the wheels are starting to fall off, and just say, “Oh, that’s just part of meditation.”*

Duration	The duration (long or short) or frequency (isolated or recurring) of an experience are factors in appraising the nature of an experience or determining need for intervention.	*At any rate, I was in this locked-open position, and by Wednesday nothing was changing. I was very wide open. And I started to get a little scared. Shouldn’t this be going back to normal by now? Shouldn’t this be reducing?* [*…*] *My hearing was very acute. My sight was very acute. Everything was very bright, and sounds were very loud. I was just in a very concentrated space all the time and it wasn’t shifting.*

Functional Impairment	Presence or absence of functional impairment is used to establish differential diagnosis or determine need for intervention. Impairments can be behavioral (e.g., disruptive behaviors), social or occupational, cognitive (e.g., impaired executive functioning), or physical.	*The phenomenology is interesting but what it’s doing to you is more interesting.* […] *You might say, “Oh they’re psychotic because they’re hallucinating.” Well, I don’t know, if it wasn’t interfering with their daily life. I’ve seen people who were psychotic, and it really is interfering with their daily life.*

Health History or Condition	The absence or presence of psychiatric, medical, or trauma history—whether known or suspected, whether active or latent, and whether treated or untreated—are factors in appraising the nature of an experience or determining need for intervention.	*My take is that either people* [*who*] *would have disturbing trajectories in their meditation—not just momentary experiences—either they are people who should not be meditating because their psychological integrity is not strong enough.* […] *So a lot of people who have had rough experiences, either they shouldn’t be doing the length or type of meditation they’re doing, or they should be encouraged to cut it way, way back. You know, I think in the meditation community there is the feeling that more is better—and more is not better. Better is better—for a whole bunch of reasons.*

Impact	Enhancing or deleterious secondary effects of the experience on the practitioner are diachronically or retrospectively identified as factors in appraising the nature of an experience or determining need for intervention. Positive impacts include ideas of spiritual or personal growth, purification, change in ethical orientation, or change in self-understanding. Negative impacts include deteriorations in primary phenomenology or negative effects from the primary phenomenology on other domains of experience.	*But, on the other hand, there were some people who knew me before and then didn’t see me for a while through that period and then saw me afterward. And I remember two of them were a pair of psychologists and I had worked with them, and they basically said, “In all the work that we’ve been doing, we’ve never seen anybody who has changed so considerably. Like, the quality of your being is profoundly different, and we don’t know how that happened, but we’ve never seen it and it’s really wonderful.”*

Phenomenological Qualities	Specific aspects of the primary experience alone are the basis for determining how to appraise the nature of an experience or whether an intervention is warranted.	*All of a sudden this tremendous amount of bliss—the jet engine feelings of bliss would come up while I was sitting and meditating. Or the white lights, or sometimes blue lights, would come up while I was meditating. It was not associated with an unpleasant feeling, or with any kind of palpitations or rushes, other than this energetic, jet engine vibration-y feeling. I could be driving, and it would come up, and it would go. At one point, I did ask* [*my teacher*] *about it and he said, “Don’t worry about it, don’t pay attention to it—it’s just a nyams.”*

Teachers’ Skills or Resources	Teachers’ ability or inability to intervene skillfully based upon their degree of training or expertise, or the resources available to teachers determines how an experience is appraised or need for intervention. Teachers’ skills refer to the perceived ability or inability of teachers to intervene successfully in the meditation-related challenge based upon their formal training or expertise either in meditation or in clinical psychology / psychiatry. Teachers’ resources refer to the amount of time teachers have to dedicate to a meditator or to additional support staff available on site who can be brought in to assist teachers in the management of a meditation-related challenge.	*When I went home and the kuṇḍalinī started*, [*my teacher*] *started realizing she was way out of her level of expertise—that she, in 30 years at the monastery, had never had a student who had gone through something like this.*

In medical, psychiatric, and psychological contexts, differential diagnosis is often made for the purposes of determining treatment. Similarly, in the VCE study many meditation practitioners and teachers made decisions based not on the nature of an experience (i.e., whether or not it was a “religious experience”) but instead on what type of response that experience warranted. We defined an “intervention” as any time a practitioner chooses or is instructed by their teacher to engage in some technique or strategy other than continuing the same meditation practice as a means of addressing a meditation-related challenge. Thus, a criterion was coded and included in this analysis if any of the following principles applied: (1) A contrasting differential diagnosis (e.g., the experience was *x* and not *y*) is made, along with one or more criteria justifying that decision; (2) Reasons were given for why a particular attribution was made, whether religious or psychopathological, even if no contrasting attribution is explicitly identified; (3) An intervention was made according to certain criteria, even if appraisals of the nature of the experience were not provided (e.g., because of criterion *x*, the practitioner or teacher engaged in intervention *y*); (4) A criterion was identified but was rejected as not valid, important, or useful for determining either differential diagnosis or need for intervention. Subsequent use of the term “differential diagnosis” in this paper should be taken as a shorthand for appraisals of either cause and meaning or determining need for intervention.

Interview transcripts were coded using NVivo 12 software. A minimum of two and in some cases all three coders (DC, JL, and NF) independently coded each transcript. Any and all discrepancies concerning whether or not to code a passage according to the principles delineated above, or which criteria category or categories should be applied were discussed by at least two coders until consensus was reached.

## Results

The following sections summarize the eleven criteria for differential diagnosis as discussed in the VCE data set. See Table 2 for how each criterion was operationalized and for additional examples.

### Phenomenological Qualities

The present analysis identified a wide range of experiences—55 of 59 phenomenology categories from the VCE study—that were appraised as expected but extraneous dimensions of contemplative development, signs of progress, and/or indications of potentially concerning psychopathology. Buddhist meditation traditions have rich oral and textual teachings detailing various conceptions of contemplative development. It is beyond the scope of this paper to cover the full range of phenomenology identified within Buddhist traditions as normal if not normative stages of the path. This section focuses on select unexpected and challenging experiences and how they were appraised by practitioners and teachers in different Buddhist traditions.

### Theravāda

Practitioners and teachers in the Theravāda tradition used a range of emic, or tradition-specific, “insider terms” to interpret experiences. One key hermeneutical framework in this tradition is the “insight knowledges” (Pali *vipassanā-ñāṇa*)—a sequence of experiences and associated insights into key Buddhist concepts. Multiple practitioners and teachers described the stages of “knowledge of arising and passing away” (Pali *udayabbaya*-*ñāṇa*) and “knowledge of dissolution” (Pali *bhaṅga*-*ñāṇa*) as progressively deeper insights into impermanence, which they frequently associated with positive affect (“everything was buzzing and everything was amazing”), somatosensory changes (“my body just exploded into fragments”), and perceptual changes (“everything is dissolving in front of me”). In the progress of insight, the knowledge of dissolution is expected to be followed by the “knowledge of appearance as terror” (Pali *bhayatupaṭṭhāna-ñāṇa*), and multiple Theravāda teachers and practitioners associated experiences of fear or terror with this stage, which they often took to be an “inevitable” part of progress on the path. However, not all experiences of fear were understood to be necessarily related to this insight. Some teachers indicated that it required discernment to distinguish ordinary fear, or “garden variety neurosis” related to “real-world things,” from this normative stage of insight knowledge. The latter involves fear that originates in the experience of dissolution and the impermanence it reveals: “the fear when you realize that the way you are perceiving the world is incorrect, and that everything is changing, there is nothing substantial there.”

Theravāda teachers identified other experiences that also required cautious discernment or monitoring. One teacher in the tradition of Vipassanā taught by S.N. Goenka explained that the term *saṅkhāra* is used to refer to “a pattern of reacting with craving or aversion to any sort of mental or physical stimulus.” A central practice in this tradition involves “survey[ing] the body in a systematic way,” which is believed to allow karmic “impurities” to arise, and then to “physically experience the *anicca*, or impermanent quality, of all of those sensations with equanimity.” One teacher stated that it is “extremely common” for this to involve experiences related to previous trauma. While many were able to work with traumatic re-experiencing in the context of practice, others were not and became “just totally decompensated to the point where it was a real crisis.”

Experiences of visual lights were often appraised as a *nimitta*—a visual image that is expected to arise as the result of successful concentration practice—which one teacher explained “you are not directed away from”; rather “you work with it in a certain way.” However, this teacher was skeptical of other visual experiences, stating that “the Buddhist tradition in particular tends to have a fairly negative take on visionary experiences. Like, if you start having any kind of visions or hallucinations whatsoever, it’s a *big* red flag. And teachers often will direct you to get rid of them.” Similarly, one practitioner meditating on a Theravāda retreat experienced an auditory hallucination of a “huge bell,” which was followed by opening her eyes and finding that “the entire room is completely gone,” after which a “blue Buddha, about three inches high” “just came zooming right in front of me.” In response, she thought, “Oh great, I’m now having hallucinations! This is totally not okay with me.”

### Zen

Teachers and practitioners in the Zen tradition employed a different range of emic terms to describe their experiences. One key term is *makyō*, which refers primarily to perceptual distortions such as illusions and hallucinations. Unlike the Theravāda subjects described above, one practitioner who described her *makyō* as “a hallucination” was told by her teacher that:

*Makyō* is very encouraging. But, you know, when you translate it literally, it’s translated as ‘devil’s dream’ because it’s seductive and you think, ‘Oh, I want to make that happen again!’ and, ‘How can I find that?’ or, ‘Oh, aren’t I so great?’—and it seduces you. And you just let it go. Just let it go. It’s nothing.

So, although *makyō* are not encouraged, neither are they signs of psychopathology or indications that intervention is warranted.

However, there are concepts within the Zen tradition for experiences indicative of imbalances in meditation practice that warrant additional intervention or support. One Zen practitioner was told by a mentor that his chronic challenges with uncomfortable somatic energy, thermal changes “where my head was very hot and my lower extremities were very cold,” constant “tenseness in my solar plexus,” and frequent “panic attacks” were likely the result of “Zen sickness,” a condition described perhaps most famously by the 18th century Zen master Hakuin. The above practitioner who reported Zen sickness ultimately found somatic treatments such as acupuncture and even the use of medications to be helpful interventions. Thus, while some experiences in the Zen tradition were valued, and others ignored, there were also certain experiences that warranted treatment with methods beyond meditation practice.

### Tibetan

Similar distinctions were found among Tibetan Buddhists. The Tibetan term *nyams*, described by a practitioner as “ephemeral meditation experiences,” was associated with a wide range of phenomenology. A teacher referred to certain “benevolent *nyams*” such as “seeing Buddhas or joining into paradises or feeling incredible gratitude or love,” but indicated it was something one is “not supposed to get attached to.” Other experiences characterized by teachers or practitioners as *nyams* include: anger, paranoia, sadness, and grief; a “blowing out of proportion” of existing emotional tendencies; hearing voices; seeing lights; feelings of bliss; becoming sick; and “feeling you’re being eaten up by demons.” Teachers and practitioners regularly referred to *nyams* as a “completely natural” part of contemplative development. They are “not the point of the practice,” but a “sign of things changing and working.”

As with the Vipassanā tradition of S.N. Goenka, in the Tibetan tradition the re-experiencing of trauma could be appraised as related to karmic purification. One practitioner engaging in Tantric preliminary practices (Tib. *sngon ’gro*) vividly uncovered a history of childhood sexual abuse that had previously been suppressed. Debilitated by flashbacks, she found it difficult to continue to practice. She was told by another practitioner that her experiences were “just part of the purification,” and that “if you’ve been keeping any secrets from yourself, they are going to come up now.” She felt she was not “well supported” by her community, as their feeling was: “You should just be doing your practices and let this other stuff go.”

Comparable to the notion of Zen sickness, in Tibetan Buddhism some physiological changes are understood through the framework of disturbance of the flow of “energy” or “wind” (Tib. *rlung*) through the “subtle body.” While meditation can cause wind to flow in a way that is believed to “purify” constrictions or “knots” in the subtle body, meditation can also cause it to flow in a way that Tibetan medicine recognizes as a disorder. One practitioner described his “wind” getting “stuck” in his “heart center,” which caused him to feel “wired and even dizzy” and left him with a “racing” mind. This required intervention, including stopping meditation, taking Tibetan medicine, and changing his diet.

### Appraisals Imported From Other Traditions

In some instances, practitioners departed from the emic concepts within their tradition by interpreting certain somatic phenomenology—specifically experiences of “energy” and involuntary movements—as indicating an experience of *kuṇḍalinī*. Although a seminal dimension of Indian Tantric Buddhism, *kuṇḍalinī* is not a normative part of contemplative development in many other Buddhist traditions. In particular, the language of “*kuṇḍalinī* awakening” reported by practitioners and teachers in the VCE study was likely drawn, directly or indirectly, from the influential work of Grof and Grof (2017), which proposes *kuṇḍalinī* awakening as a key type of spiritual emergency. Although multiple practitioners found that this framework matched their experiences, they did not always follow the spiritual emergency approach that encourages allowing the experience to run its course without medication-based interventions.

Modern Buddhists have also adopted the term “dark night,” which originates in the writings of the Spanish Catholic mystic St. John of the Cross and there refers to a period of absence from the presence of God. Theravāda meditation teacher Kornfield (1993) used this term to characterize the more challenging stages of the progress of insight, especially the stages referred to as the “knowledges of suffering” (Pali *dukkha-ñāṇa*). Multiple practitioners and teachers in the VCE study from across Buddhist traditions referenced the “dark night” as a shorthand either for challenges associated with the knowledges of suffering in particular or for meditation-related challenges in general.

### Problematic Types of Experience

Some experiences were consistently assessed as problematic, rarely identified as intended or expected stages of the path, and typically taken as indicating need for intervention. For example, teachers consistently regarded suicidality as a problem that clearly required intervention. One said, “if somebody were to come in and say: ‘I think I’m gonna kill myself today,’ that would be something that would be, obviously, a big red flag.” For those practitioners who reported suicidality, it was invariably understood as a problem, and was often addressed by seeking psychological or psychiatric help, including hospitalization and medication.

Also subject to further scrutiny were practitioners reporting phenomenology closely associated with mania and psychosis, such as loss of sleep and appetite, exaggerated positive affect, delusions, paranoia, and in some cases hallucinations and disruptive behaviors. For example, one Theravāda teacher intervened when he saw a student “get wound up in an ecstatic way” on account of intensive practice as well as not eating or sleeping. Such experiences, sometimes in conjunction with but also irrespective of the presence of additional criteria, were commonly identified by experts as warranting an intervention, which could include being taken off retreat and placed into psychiatric care.

Finally, even if a specific experience was appraised as an expected stage of the path within a Buddhist tradition, when other criteria for differential diagnosis are present, the experience could be seen as warranting intervention nevertheless.

### Distress

As mentioned above, phenomenology that involves some degree of distress is considered expected or even an explicit part of the path in some cases, such as the “knowledge of appearance as terror” stage in the Theravāda progress of insight. Nevertheless, some Theravāda teachers differentiated expected examples of such feelings, e.g., “I feel terror, and I sit there in the room” versus “I’m fidgety and feel, all of a sudden, terror comes over me, and I have to run out of the room.” Teachers voiced concern about distress when an experience became “overwhelming” and beyond the “capacity” of the practitioner to hold. Although this might warrant special care or intervention, it did not necessarily mean that the experience itself should be seen as pathological. One Theravāda teacher explained that intense experiences associated with the “arising and passing away” stage “can be distressing for people because they’re not used to it.” This teacher also explained that “most of the time when people come with an overwhelming experience, I would put it in the category of the *saṇkhāra*”—that is, a mental disposition that is to be worked with in the context of practice. However, distress could be compounded by duration, as this teacher also acknowledged that if “they continue to feel overwhelmed for days on end, that’s too much,” and they should be removed from the retreat. One clinician and meditation teacher looked to physiological signs, such as restlessness, breathing changes, dissociation, and ability to maintain eye-contact, for indications that “No, this isn’t part of meditation.”

Similarly, some practitioners interviewed in the study reported feeling “scared,” “freaked out,” “distraught,” “overwhelmed,” or “destabilized” by meditation practice. Although some instances of distress were framed in reference to expected stages of the path, it was more typical among practitioners for distress to lead to some sort of intervention such as changing meditation practice, stopping meditating, engaging with grounding activities, seeking psychotherapy, or taking medications. Some practitioners also took distress as the basis for a change in the appraisal of their experience or as a key criterion in the determining of its meaning or value. Such appraisals ranged from general concerns about “going crazy” to more specific psychiatric language such as having “panic attacks.” One practitioner reported asking her teacher about a distressing change in sense of self because “I didn’t know if I was having like an enlightenment experience or something. And she said, ‘No, it wasn’t enlightenment because you were suffering too much.’ So I thought that was a very powerful statement.”

In summary, while some distress was normalized as part of the path, teachers and practitioners alike also looked to distress as potentially indicating a need for intervention, and in some cases as the basis for an appraisal concerning the nature of the experience.

### Control

Many teachers became concerned if practitioners were unable to follow practice instructions or were unable to “titrate or contain” their experiences. One meditation teacher and clinician described a “big difference” between practitioners who are able to remain “grounded and confident” in the presence of novel experiences, and others for whom their experience is “like a hot air balloon that’s just slipped its moorings and is starting to float off into the sky without any direction. […] And they don’t know how to operate this hot air balloon.” Conversely, one teacher acknowledged that there is a stage of practice in which “the practice really takes over and we recognize we’re not in control. And that can be very unsettling,” leading to fear and anxiety—but this was not an indication that something is wrong or that an intervention is needed.

Similarly, several of the meditation practitioners interviewed mentioned that certain meditation experiences—especially those associated with somatic energy (Skt. *kuṇḍalinī*; Tib. *rlung*)—were inevitably going to persist beyond the control of the meditator. One Tibetan Buddhist practitioner explained that

what happened was a lot of this *kuṇḍalinī* got stirred up in this first retreat. […] Once you kind of awaken that, or if you intentionally go after it—you have no control over it! And it just does what *it* wants to do. And it can really be incredibly painful, incredibly disruptive. You know, it can absolutely cause psychosis.

Multiple practitioners were encouraged to stop meditating when they developed ongoing energy experiences that could not be directed or managed intentionally.

Other types of emotional, cognitive, and behavioral experiences could also develop beyond the practitioner’s control. One Tibetan Buddhist practitioner reported that the absence of control over her experience could be viewed through either a religious or a biomedical perspective:

Everything is very much beyond my power. It’s like if a stream of grace ripens, I’m in that. If a hell dimension opens, I’m in that. And I’m just in it till it exhausts itself, and I still don’t have any power to engage or accomplish or effort in anything. […] I contemplate what it all means spiritually. Am I going through a crazy purification-slash-initiation or is it just a really intense illness?

In severe cases, loss of control over one’s experience could result in behavioral changes that teachers or the meditation community considered to be inappropriate, disruptive, or concerning. One Zen practitioner reported how a loss of control leading to disruptive behaviors led to him being asked to leave the monastery:

The bell had rung to end the sitting meditation and to begin the walking meditation, and I was in such a deep meditation I didn’t want to interrupt it, and I didn’t move. And so one of the nuns came up to me to prompt me to go out and start walking meditation, and I roared at her—like a lion’s roar. And this was just mania. And I was immediately moved [out of the meditation hall]. […] And I ended up going into this hysterical laughing. And one of the monks came in and told me that…he said, “[practitioner’s name], this is not Zen. You have to leave.” And he walked me to the car. He said, “When you’re in control of yourself, you can come back. This is not Zen.”

While the practitioner in this case ultimately viewed his experiences as the result of a meditation-induced manic episode, the next passage illustrates how in another community a similar loss of control and outburst elicited an intervention but was ultimately given support within the retreat setting:

I got into a pretty big trance state, and that seemed to be all fine with everybody there until somebody got worried because I fell back, and a retreatant came over very anxious. […] And she came up and interrupted me, and so I didn’t have much self-control and I just yelled out. […] But when I yelled out, then that’s when they took me out of the shrine hall. […] Then two folks who were part of the protection *maṇḍala* sat with me and were working with me to help get more grounded.

Loss or lack of control ranged from an expected dimension of certain meditation experiences, to the basis for a change in or questioning of appraisals, to a criterion for determining need for intervention. Like many criteria, it may be essential to consider control in relation to the specific phenomenology as well as to behavioral changes, which may be given greater or lesser degrees of tolerance in different meditation communities.

### Duration

Duration was another criterion the importance of which varied considerably depending on the type of experience in question. Certain experiences, such as perceptual hypersensitivity or mental stillness, were expected to have a prolonged time course in some situations, and other experiences, such as enduring shifts in one’s sense of self, were even viewed as explicit goals or stages of the path. Some changes, such as insomnia, might be expected in the short term but became concerning if they went on for a few days. In contrast, other experiences such as suicidality or disruptive behaviors tended to be concerning whatever their duration and were likely to be taken as indications that immediate intervention was warranted.

Theravāda teachers interviewed in the VCE study disagreed about the expected duration of the difficult stages of the progress of insight. One teacher in a monastic context said each stage shouldn’t last for more than a day; however, another teacher thought that cumulatively they could last for months or years. Two teachers agreed that they would be concerned if someone got “stuck” in the progress of insight, or if their experience in one of those stages “was not properly integrated and it developed its own dynamics and got out of hand.” Another teacher became concerned when “the fear won’t go away even if they stop meditating.” The same was true for somatic experiences—such as pain or nausea—which for one teacher would be acceptable within the context of a meditation session, but would be more likely to be indicative of a physical or medical problem if they endured much beyond the end of the session.

Meditation practitioners also exemplified how duration as a criterion for differential diagnosis depended on the phenomenology in question. As mentioned above with respect to control, some practitioners reported experiences of somatic energy that lasted for many years, and which they took to be within the realm of what would be considered normal by at least some meditation traditions, even if not their own tradition. However, as with teachers, multiple practitioners became concerned when experiences they expected to be transient persisted long after the end of periods of formal practice and interfered with daily life. For example, a Theravāda practitioner reported a meditation session that was “particularly deep; I was losing sense and awareness of my body.” The experience “just kept continuing even after meditation” and led to him to seek out help from his teacher to try to re-ground himself in his body. Similarly, a turning point for a Tibetan Buddhist meditator who had been on an extended retreat practicing *śamatha* (a concentration technique) was when she “attempted to stop meditating and these mechanisms that I’d practiced during meditation continued anyway.” Finally, another practitioner was very confused because

lots of people have energetic experiences where they feel voltage. Lots of people have trauma memories come up. That’s not unusual, that’s a part of the practice. […] I saw a lot of my peer group move through these experiences, and I have not moved through them, and I don’t understand. […] Some people pass through in one meditation period, some people it’s a longer thing. But, for me, I’ve been semi-impaired for a couple years now, and that’s what I don’t understand.

Thus, as in other diagnostic systems, duration often interacts with control or functional impairment when determining an appraisal or need for intervention.

### Impact

Although there were a few exceptions—such as the statement above that *kuṇḍalinī* could lead to psychosis—assessments of impact typically took positive, beneficial impacts as indications of a spiritual or religious experience and negative, deleterious effects or deteriorations as indications of psychopathology or a need for intervention. One teacher differentiated depression from the “dark night” by stating that only the latter would result in “breaking out into equanimity.” Other examples of positive impacts include behavioral or interpersonal changes assessed retrospectively. One practitioner who had long-term challenges associated with somatic energy reported that “ultimately this whole process anchored me in loving-kindness,” such that people he knew told him “the quality of your being is profoundly different” than it had been previously. Other aspects of contemplative development, such as beliefs about purification, were also associated with reports of positive impacts. One practitioner engaged in *vipassanā* practice characterized his experience using the tradition’s rhetoric of “the removal of impurity”: “when this stuff dissolves, I feel this relief for a while until the next thing comes up.” Another practitioner in the same tradition described how the goal was to “work through these karmic issues that were stored in the body,” which was associated with “physically a feeling of being lighter.”

Conversely, experiences with negative impact were typically taken as indications of a need for intervention and could also serve as the basis for appraising an experience as indicating potential psychopathology. Situations that tended to signal a need for intervention included deteriorations, or the experience worsening or intensifying over time, and impacts spreading from the primary phenomenology to affect other dimensions of experience. For instance, one practitioner became concerned when he “hardly had any sleep that night because of this experience [of distortions in his sense of embodiment] continuing but seeming to get much worse as the night progressed.” Teachers and practitioners alike described how practices that would be beneficial in one context or to a certain degree could have negative impacts. For one teacher, “The clinical issue arises when these things [somatic focus and self-scrutiny] go off the rails and become uncorrected in their impact.” Serious deteriorations could even result in functional impairment.

### Functional Impairment

Practitioners and teachers alike held mixed views as to whether or not and in what contexts functional impairment could serve as a sufficient criterion for differential diagnosis. One teacher made his differential diagnosis explicitly on the basis of functioning, granting this criterion a paramount importance superseding other criteria and concerns, stating, “My definition of mental illness is largely functional.” By contrast, another teacher identified two types of impairments that could be associated with a stage or depth of practice. The first was “the standard spiritual emergency where people have *kriyas* [involuntary movements] and energy things and a lot of fear,” and the second was other people who come out of retreat and “don’t know how to get all their ordinary functioning to work again for a long time.” Both cases were considered possible outcomes of intensive practice that warranted additional supervision or guidance, but would not necessarily be an indication of psychopathology.

For practitioners, the severity of their functional impairment was often an indication to others—whether teachers, friends, or family members—that intervention was necessary. Minor impairments in functioning could sometimes be resolved through management strategies such as discontinuing practice and grounding activities, and these did not necessarily indicate potential psychopathology. So too, even severe and ongoing functional impairment could be associated with both spiritual and psychopathological appraisals. For example, one practitioner who had year-long functional impairments in her emotional life in particular described her experience both in terms of a bipolar episode and a *kuṇḍalinī* awakening.

Many driving-related incidents were indications of concern and a need to become more grounded and in control of one’s experience, but some were still interpreted as a normal, if undesirable, consequence of integration following retreat. One practitioner wasn’t sure he was safe driving because of having the “sensation of my car moving forward, but somehow I was moving backward with the scenery. […] I think that was some kind of expansion and contraction awareness that I had.” Another reported “looking at a stoplight and seeing the color come up, but then having to think, ‘But what does that mean again?’ and having to remember whether it meant stop or go.” A more serious incident was when a practitioner hit seven parked cars due to perceptual distortions in distance, a situation that ultimately led to psychiatric hospitalization.

Still, impairments that impacted behavior and interpersonal communication were often viewed as serious and could result in hospitalization. Two practitioners were hospitalized when they stopped communicating with others and became non-responsive. Many other practitioners—both those who were hospitalized as well as some who were not—sought the use of medication as a means of resuming their normal functioning. One practitioner said of his experience: “I’m stuck between maps, basically. But the one that I’m using to be a functioning person right now is definitely a psychiatric one that includes psychopharmacology.” The need for hospitalization and medication often raised questions for teachers about possible pre-existing or co-existing conditions as indicated by a psychiatric history.

### Health History or Condition

Health history or condition was one of the more controversial criteria discussed by subjects in the VCE study, with teachers and practitioners disagreeing about the scope, relevance, and reliability of this criterion for differential diagnosis. At one extreme, multiple teachers made sweeping statements in which psychiatric history was the primary explanation for meditation-related challenges. For example, one teacher stated that “I’m really not aware of situations where people take up meditation and something about the meditation causes them some difficulty that wasn’t pre-existing. […] It’s always someone who has that pre-existing difficulty.” Similarly, another stated that “honestly, the ones I’ve seen where I would say there is quote-unquote ‘impairment’ are people that have mental health issues.” Another teacher identified trauma rather than psychiatric history as the basis of meditation-related challenges. He expressed concern that for people with trauma histories certain types of practices would be “almost immediately destabilizing” because “they don’t have a very well-formed or stable psyche to begin with because of various kinds of childhood trauma.” Other teachers noted the challenges they face when working with “people who didn’t know they had preexisting conditions until they started meditating,” such that “people all of a sudden start having memories that they didn’t have before.” One response was to “normalize the challenges but also take them very seriously at the same time.” Another teacher stated that “certainly I think that there’s an interaction with meditation that may precipitate [a mental health issue] a bit sooner. […] Frankly, I think it’s a stretch to say, ‘Oh, it has absolutely nothing—there’s no relationship to the meditation at all.”’

Practitioners tended to be more critical of psychiatric history or trauma history as a valid and reliable criterion for differential diagnosis. A few practitioners reported that teachers they worked with tended to assume a psychiatric or trauma history when they didn’t know how to proceed, which practitioners found presumptuous and confusing. One practitioner explained:

I remember, they were like, “Well, it seems like there might have been some sort of trauma—did you have some sort of trauma in your life?” And I haven’t thought of any. I can’t think of one thing. *This* was the most traumatic thing that happened in my life!

Other practitioners rejected this criterion as a complete explanation of their experience: “if somebody told me that all this is just a symptom of bipolar and was rooted in some deeper relational disruption from childhood with a caregiver, I’d say, ‘No, I don’t think so.”’

Some teachers also believed that it was difficult to rely on psychiatric history as a means of differentiating psychopathology from normative spiritual experiences, explaining that a past episode of illness is not a necessary or sufficient reason for a manic or psychotic episode to occur during a meditation retreat. Although people with a past history “seem extremely prone to having a manic episode on or soon after a course,” at the same time “certainly we’ve had people who never had a manic episode have a manic episode on a course.” Similarly, another teacher and clinician summarized his view as follows:

anyone under sufficiently adverse physiological and psychological threat can have a psychotic episode. So I don’t think it’s an either/or. I think those predisposed are on a spectrum, and certainly in my experience people with a prior history of severe mental disorders are more likely to have severe mental dysfunctions as a result of meditation practice and particularly as a result of intensive retreats. Now certainly I’ve seen some people who haven’t had previous psychosis have transient psychotic episodes.

### Circumstances of Onset

One practitioner in the study explicitly stated (and many others implicitly believed this as indicated in the way they responded to difficulties) that because her challenges were caused by meditation, the way to address them was also through meditation. As a teacher interviewed in the study put it:

By and large we view pretty much everything that comes up on retreat as being part of retreat. […] We frame the entirety of the practice as purification of mind—the idea being that whatever is arising is part of what’s wanting to be purified, what’s coming up organically.

However, another clinician and meditation teacher explicitly rejected this criterion as relevant, stating:

Where do you draw the line and what’s in the territory in between? I’m not really sure. But I do think that there are people who will go on retreat and it may trigger, really, a psychotic kind of experience for them, and it doesn’t necessarily mean that it’s a spiritual experience that they’re having.

One other teacher used the absence of other environmental or life stressors as an indication that challenging emotional experiences should be viewed as part of the progress of insight rather than as indicative of depression. Overall, very few practitioners or teachers in the VCE study put forth circumstances of onset as a viable overarching criterion for differential diagnosis or determining need for intervention.

### Critical Attitude

Critical attitude was almost exclusively associated with assessing hallucinations, delusions, disorganized thinking, and disruptive behaviors—phenomenology which led to practitioners (and more commonly teachers) needing to determine whether their experience indicated potential psychopathology. One practitioner reported being unable to determine whether she was located in her body or in her reflection in the mirror. While she was concerned that she had “really gone crazy,” her husband reassured her that “You know it’s an altered state. If you were actually crazy, you would have inhabited that state.” Another practitioner became concerned when he could no longer tell whether or not he was in a dream, and consequently he sought help and reassurance from his teacher. One practitioner, in differentiating visionary *nyams* from hallucinations, said that with *nyams* “you are not completely identified with the experience.”

Other practitioners and teachers alike looked to behaviors, especially speech, as indicative of a meditator’s degree of “coherence.” One practitioner explained, “People who were going through spiritual emergence could talk incredibly precisely and cogently about their crazy-seeming experiences, whereas somebody who was mentally unbalanced wouldn’t be able to do that.” When critical attitude was ambiguous or weakened, this could be cause for concern and potentially intervention. One practitioner reported feeling the following way:

I was sufficiently solid in my ego structure that I knew that there was this quality about this that was fucking crazy. But there was also this part of me that was kind of believing it, too. And it was that quality that was most unsettling. […] And I do think it was kind of a meditation psychosis.

Another practitioner reported the loss of critical attitude as a turning point in how he appraised his experiences: “I just lost the ability to realize that those things were just mental images. And so I started having what I understand to be ideas of reference.” Finally, a clinician and meditation teacher summed up critical attitude as a criterion very well when he stated that when working with practitioners reporting something that sounds like auditory or visual hallucinations, the key feature is “how they relate to those and whether they relate to them as real or not and whether they can differentiate between what’s consensus reality and what’s not consensus reality.” The presence or absence of critical attitude was sometimes what differentiated whether an experience was appraised as a vision versus a hallucination, or as paranormal versus delusional.

### Cultural Compatibility

Buddhist meditation traditions have many frameworks for identifying experiences that are viewed as a normal if not normative part of the path of contemplative development. For example, one practitioner reported that he viewed the various experiences he was having on retreat—“pain [or] tension, or heat, or cold”—as normal because the Vajrayāna theory of the subtle body “makes sense” of those experience as instances “in which the flow of wind through the subtle body have been constricted.” Challenges associated with states of meditative absorption (Pali *jhāna*) were also assessed in relation to cultural norms and values. For instance, one practitioner came to identify a pattern of entering into extended “trances” during long periods of meditation practice. Despite being placed into psychiatric care twice, her experience was normalized by a neurologist “as going into meditative absorptions really quickly, and then having trouble coming out.” This led her to wonder whether her Native American heritage would say, “‘Oh, it’s related to shamanic trances.’ So it sort of depends on the perspective of the person.” By contrast, multiple practitioners described how they came to understand their way of practicing in relation to difficult emotions as a “dissociative space” or having “a lot of difficulty actually observing physical sensations” on account of “being dissociated to those exact sensations.” One practitioner had to learn how to work against a tendency of “going to those out-of-body places” She explained this process in relation to a trauma model: “All of these things, in hindsight, were inhibitory to my nervous system, were certainly not helpful to trusting myself, and served to drive me further into ‘freeze-disconnect-spaciousness’ and fed a kind of psychological dependency—things that on the outside made me appear to be a ‘good Zen student.”’

### Negotiating Multiple Worldviews

Teachers sometimes framed experiences in ways that drew upon both Buddhist and psychological models for understanding experience. One teacher thought that “repressed traumatic experiences may come” during concentration as a consequence of “breaking the barrier between the conscious and the unconscious mind.” However, the process of differential diagnosis could also highlight differences in or conflicts between the cultural frameworks of Asian teachers and Western practitioners who were their students as well as mismatches between Buddhist and psychological or biomedical epistemologies. One practitioner explained how when she was going through a meditation-related challenge, a friend of hers offered her books to read to help her to understand her experience, explaining that “In America, we might think the person is crazy, but if you were in India you’d probably be supported by a family and have your own cottage in the back! Your own meditation cave!” Another practitioner felt that her Tibetan teachers “really didn’t understand Western psychology.” Although she was a nun in a Tibetan lineage, she prioritized her own cultural background in interpreting her meditation-related challenges: “I chalk it up to the brain chemistry that just went to hell in a handbasket for me.” One teacher also noted that working in communities with multiple worldviews presents difficulties for establishing a differential diagnosis. This teacher prioritized what she thought was most compatible with the worldview of the person undergoing the experience and of the community in general:

And other people have different points of view. I had another nun who sent me this one person who I think is psychotic and she tells me, “Well, this is spirit harm.” Okay, you can call it and label it whatever you want.

In some cases, practitioners and meditation communities could not come to agreement on the appraisal of an experience. One Theravāda practitioner was initially not given a framework for understanding her experience of somatic energy. She later had a number of people tell her that she had a “*kuṇḍalinī* release,” which she found helpful. However, when she called the retreat center where she had been practicing to explain her current appraisal, “they said they don’t believe in *kuṇḍalinī* releases.”

In other instances, the dynamic between biomedical or psychological and Buddhist frameworks was more implicit. One practitioner reported how for many years he was resistant to a mental health diagnosis because “I always just thought I needed more practice.” Thus, general expectations about the nature of the path and the types of experiences that can be held in the context of meditation practice can affect decision making about how to appraise or whether to intervene in an experience.

### Socially Disruptive Behaviors

Violations of socially accepted behaviors were commonly seen as indications of a problem warranting some form of intervention. In some instances, even minor violations of ritual protocol, such as turning sideways in the middle of a meditation session in a retreat hall, or entering the meditation hall before the appropriate time, could indicate something unusual in a practitioner’s behavior. By contrast, one practitioner—who herself experienced intense involuntary movements in the midst of a group meditation session—reported that in her community “there’s a lot of room for people having their experience. And I think there’s some expectation that people are going to have weird experiences and, as long as it’s not too disruptive, there’s a space for holding whatever people need to go through at a somatic level.” Implicit in many cases of disruptive behaviors are decisions about the degree to which teachers have the skills or resources to manage such experiences.

### Teachers’ Skills or Resources

Teachers’ skills and resources was typically invoked in the context of determining need for intervention—a decision that often superseded concerns about the nature of a given experience. Several teachers described how they evaluated the nature of a meditation experience based upon their cumulative training expertise or “intuitive” knowledge. One teacher relates how his meditation teacher training has yielded an “intuitive sense of certain kinds of processes that people need to go through.” This same teacher explained how his own contemplative practice contributed to the intuitive sense of others’ progress. Meditation teachers with dual training as clinical psychologists or trauma therapists also made “intuitive” decisions based upon “the pit in my stomach” to assess whether a challenging experience is best addressed through additional practice or through therapy. Practitioners described how their teachers recommended other types of intervention when they lacked specific knowledge of particular meditation-related challenges or were ill-equipped to respond. For example, one practitioner shared that after several months of “manic” symptoms, his teachers eventually “felt they weren’t in control of what I was doing, and they had to take me to the hospital.”

Some teachers emphasized that in order to make an accurate differential diagnosis, they needed to spend a significant amount of time with a student because “you can’t necessarily tell […] whether it’s a true spiritual experience or not if you don’t know somebody well.” One meditation teacher highlighted the longitudinal process involved in evaluating stages of the path:

I don’t feel so comfortable assessing whether someone is in the stages of insight unless I have tracked them go through it. […] Not everyone goes through the stages of insight the same way. […] A one-time report, it’s hard to know.

Another meditation teacher and clinician similarly conveyed how the differential diagnosis decision tree involves

Knowing the person over a long period of time and really seeing them through a number of different life experiences. If I was doing an evaluation in an emergency room, I don’t know that I could differentiate. […] It takes a lot of years of clinical work to get a sense—and it *is* a sense—of how these different kinds of disorders feel. […] It’s a matter of trying to make a subjective judgment here. I think it would be very hard for Dharma teachers who don’t have the clinical background to make that kind of judgment.

Other teachers agreed with this concern, making statements such as “I’m not a professional therapist or psychologist or anything like that, so I don’t feel very well-equipped to handle people with serious psychological issues” and “Well, the first clue would be: I don’t know what the hell to do with this! It’s like: okay, this is a suffering or delusion that’s beyond my pay grade.” They noted, rather, that “meditation teachers are not trained to deal with psychological disorders. Psychotherapists are trained to do that.” Similarly, another teacher stated that in his tradition in general, “we’re very cognizant of the fact that we are not mental health professionals”; as a consequence, he focused on determining whether or not practitioners can work with their challenges solely through continued meditation practice.

## Discussion

Various criteria have been proposed as a means for distinguishing between spiritual, religious, or mystical experiences and forms of psychopathology. Practitioners and teachers in the VCE data set employed all criteria identified in previous research in navigating meditation-related challenges. However, an investigation of how meditation-related challenges are assessed in the real-world context of Buddhist communities also shows that criteria for differential diagnosis are not consistently applied or universally reliable. Additionally, this study found that the range of phenomenology that prompts efforts at differential diagnosis is much larger than the oft-discussed distinctions between psychosis and mystical experiences. Part of this may have to do with the fact that much of the previous research on differentiating religious experiences from forms of psychopathology has been based upon textual sources or isolated case studies and has proceeded under the assumption that religious experiences are *sui generis* (Taves, 2009, 2020). Investigating processes of differential diagnosis among practitioners and teachers in contemporary communities reveals additional phenomenology under evaluation, ambiguities in some proposed criteria, and different emphases placed upon certain criteria in particular situations.

Many types of difficult experiences are recognized in Buddhist textual and oral teachings (Lindahl et al., 2019). Practitioners and teachers alike made reference to tradition-specific notions of difficult stages of progress or meditation-related side effects, such as the progress of insight (Sayadaw, 1965; Buddhaghosa, 1991), *makyō* (Sogen, 2001), and *nyams* (Gyatso, 1999; Lingpa, 2015). They also drew upon traditional conceptions of psychosomatic disorders such as Zen sickness (Hakuin, 2009; Ahn, unpublished) and wind disorders (Lindahl, 2017; Deane, 2019) that can be triggered or exacerbated by meditation, as well as frameworks more commonly associated with other religions or with “spiritual emergency,” especially *kuṇḍalinī* (Sannella, 1987; House, 2010), and the “dark night” (Kornfield, 1993; Lutkajtis, 2019). It could be the case that the presence of certain challenging experiences in the textual corpus of Buddhist stages-of-the-path literature in particular presumes an absence of other potentially relevant criteria such as duration, distress, control, or functional impairment. Several commonly used criteria for differential diagnosis also have important exceptions in Buddhist conceptions of contemplative development such that these criteria can be superseded by other criteria or overridden by specific views and values from within the tradition. This was evident in the varied discussions of distress, control, and duration, and although functional impairment was seen as regularly warranting intervention, this did not necessarily lead to a psychopathological appraisal. Together this shows it can be difficult to generalize about how to apply differential diagnosis in certain local contexts, as these criteria often have to be negotiated in relation to the other emic frameworks held by members of communities. Thus, while these criteria remain useful, they cannot be applied in all cases as self-evident rules for determining the nature of an experience (Marzanski and Bratton, 2002; Rashed, 2010).

Two criteria—positive impacts and a meditator’s health history or conditions—seemed particularly problematic with respect to practical decision making about meditation-related challenges. First, relying on downstream positive impacts in order to retrospectively appraise an experience as spiritual or religious does not facilitate immediate decision making about whether an intervention is warranted. One of the consequences for those who valued this criterion and framework was that further meditation practice might be recommended instead of alternate interventions, which in some instances may have further exacerbated the problem. Furthermore, the use of vague and unfalsifiable constructs such as expectations of “purification” or “spiritual growth,” also hinder the utility of this criterion. Although negative impacts or deteriorations also require a diachronic assessment, these judgments tend to be made upon observable behaviors such as ongoing or increasing symptoms, or worsening levels of distress or functional impairment.

A meditator’s medical, psychiatric, or trauma history or conditions could also function as a retrospective explanation for why some practitioners had challenging or destabilizing experiences while others did not. Teachers sometimes assumed that psychiatric or trauma histories were the primary and sometimes even the sole explanation for meditation-related challenges, although the demographic data from the VCE study does not support this strong association (Lindahl et al., 2017). Indeed, this criterion could lead to circular reasoning as well as discrimination: if challenging or unusual meditation effects that occur in individuals with psychiatric histories are always evaluated as psychopathology, then this criterion would preclude those with psychiatric or trauma history from having their challenging experiences legitimately appraised as spiritual or religious. In fact, psychiatric or trauma history and religious experiences are not mutually exclusive. Practitioners with psychiatric or trauma histories can have what they or others consider to be valid, if challenging, religious experiences; conversely, as many teachers and practitioners stated above, individuals without any prior history of mental health problems can develop meditation-related challenges appraised as psychopathology (Yorsten, 2001; Lindahl et al., 2017; Lindahl and Britton, 2019). Evaluating experiences with retrospective criteria such as positive impacts or health history also enables authorities (i.e., meditation teachers) to claim the benefits from the cases that happen to turn out well by appraising such experiences as religious and attributing those effects to the practice, while deflecting responsibility for and distancing themselves from the negative implications of the cases that do not turn out well.

Efforts to distinguish religious experience from psychopathology and to determine the need for clinical referral or intervention are inevitably negotiated in social contexts, with their own interpersonal and institutional dynamics. Therefore, articulating and applying various criteria for differential diagnosis should take context into consideration, including: (1) the needs, motivations, and goals of meditation practitioners; (2) the expertise and resources of meditation teachers; and/or (3) the appraisals of meaning and value derived from the worldviews, practice approaches, and conceptions of the path of specific meditation traditions. While appraisals of specific experiences might seem to be simple applications of criteria, they operate with numerous background assumptions that inform the decision making of both practitioners and teachers. These include conceptions of the types of experiences to be expected as normal or normative aspects of contemplative development, as well as conceptions of mental health and mental illness. These can also depend upon training, expertise, and available resources (e.g., time, support staff, etc.). Despite the increasingly blurry boundaries between meditation and mental health care, among the experts interviewed in the VCE study, meditation teachers who were also clinicians were more inclined to emphasize watching for signs of dysregulated arousal or to recruit both Buddhist and clinical explanatory frameworks, whereas many meditation teachers without clinical training emphasized the boundaries of their expertise and their hesitation in providing any form of mental health care.

Another key finding in this study is that in many instances, the focus of meditation practitioners and meditation teachers was not strictly determining the nature of a given experience as either “religious” or “psychopathological,” but a more pragmatic concern with determining whether a particular experience, regardless of appraisal, warranted additional support or intervention. Shifting the question from the either/or of differentiating religious experience from psychopathology to determining the need for some form of intervention can allow a more nuanced and flexible approach to emerge, especially in ambiguous situations.

Accurate differential diagnosis of distress related to spiritual practice is important for clinical, social and ethical reasons (Jackson and Fulford, 1997; Brett, 2002). Clinically, it can guide an appropriate response to restore health, adequate functioning, and well-being. Early recognition and treatment of psychotic illnesses may improve the long-term course. Misattributing such illness could lead to delays in treatment and increased risk of chronicity (Santesteban-Echarri et al., 2017). Socially, accurate assessment provides important information about the impact of particular religious practices and settings that can guide corrective actions and the decisions of others seeking spiritual instruction. Too often, spiritual practices are seen as panaceas, and negative effects are downplayed or ignored. Any practice powerful enough to effect major changes in experience and life-orientation also has the power to disrupt adaptation. Ethically, such potential harms need to be recognized and prevented or addressed. At the same time, it is crucial to avoid pathologizing valuable processes, which may be painful and disruptive, but yield desired growth and insight.

Distinguishing personally and spiritually valuable experiences from illness and affliction that require medical and psychological intervention is challenging but becomes especially difficult in settings where there are conflicts of interest. The social and professional positioning of the appraiser often determines which frameworks are applied and how (Helderman, 2019), and this may not always be in the best interests of the practitioner. When an appraisal of a practitioner’s experience is used to protect the interests of the teacher and organization, there is the potential for great harm to the individual. In contrast, when differential diagnosis is person-centered and the practitioner’s values and goals are explored as part of the assessment process, some of these problems can be avoided (Kirmayer et al., 2016). Making sense of the individual’s experience also requires systematic attention to the social and political contexts in which it occurs. A truly person-centered differential diagnosis would take seriously the criteria that the meditator would use or want used, even if this conflicts with the personal views of the meditation teacher or the religious tradition (Lindahl et al., 2019). However, challenges to the person-centered approach arise when considering more extreme cases, such as may occur with psychotic delusions or mania, in which practitioners may not be able to evaluate the degree to which an experience is in their best interest or aligned with their values. Thus, it may be essential to take into consideration not just the views and values of the meditator, and their own capacity think through the consequences of their experience, but also their social relationships within and beyond the meditation community.

Multiple possibly conflicting worldviews or value systems may be held by contemporary Buddhists in the West who find themselves caught between biomedical and religious notions of well-being and purpose. Given the diversity of meditation traditions and their various relationships to the epistemologies of science and medicine in the West, we would expect variability in how communities delineate and employ the criteria for differential diagnosis. Taylor and Murray (2012) contend that clinical interventions for anomalous experiences are more effective when they accord with “the understanding and meaning relevant to the culture of the individual having the experience,” and in cases where there are conflicting explanatory frameworks, acknowledging the individual’s preferred cultural framework(s), whether religious or biomedical, is beneficial (p. 11). Interestingly, Taylor and Murray (2012) consider biomedical explanations as part of a “psychiatric culture” comprised of particular “narratives, behaviors, and rituals” (p. 13). Similarly, Kaselionyte and Gumley (2019) show how extreme mental states associated with meditation can be situated within either “biomedical” or “alternative” discourses (or in some cases both), each of which has its own biases.

Cultural background knowledge not only provides interpretive frameworks that influence how individuals respond to a challenging experience, but also includes embodied skills and environmental affordances that may shape any experience from its inception (Cassaniti and Luhrmann, 2014; Kirmayer and Ramstead, 2017). Haslam (2005) has identified some common modes of explaining psychiatric symptoms or problems used by lay people that invoke medical, psychological or moral frameworks. To this list we might add religious or spiritual modes of explanation. Each mode of explanation influences coping and help-seeking. Of course, mental health interpretations of experiences are also cultural systems of meaning, and they act to change the nature of experience through what Hacking (1999) has called “looping effects.” Meditation teachers, communities, and popular media are all part of social systems that articulate and reinforce particular norms as well as modes of experiencing and coping with challenging experiences. Together, these arguments suggest that challenging experiences become pathologized or normalized only under certain conditions; under other conditions, the same or similar experiences may instead be contextualized by recruiting non-medical, cultural frameworks, such as religious frameworks. Systematic assessment of the multiple cultural frameworks employed by an individual and those close to them provides a basis for person-centered care (Kirmayer et al., 2016). Recognizing psychiatry as a distinct culture (or, more accurately, subculture) allows mental health professionals to think in terms of the negotiation of cultural differences when a meditator engages with biomedical treatments while also holding another culturally salient explanatory framework. Ultimately, greater dialogue among psychologists, psychiatrists, and meditation teachers would be useful for understanding the complexity of these issues and for determining appropriate interventions for those with meditation-related challenges.

### Limitations and Future Directions

The qualitative retrospective design of the current study and the lack of systematic inquiry into decision-making processes limit the generalizability of findings and our ability to establish the accuracy, utility, and reliability of particular criteria. The VCE study is based upon a non-representative population of Western Buddhists purposely selected for their ability to report on meditation-related challenges, their interpretation, and their management. Thus, these data may not generalize to other religious traditions, to mindfulness-based interventions, to clinicians without backgrounds in mindfulness or Buddhism, or to Buddhist communities in other cultures. Future studies should systematically explore differential diagnosis criteria for different types of experiences and investigate the degree to which there is a hierarchy of criteria, where certain criteria are reliably superseded by others. Prospective studies with longitudinal follow-up and quantitative measures can establish which criteria are predictive of outcomes.

## Conclusion

The differential diagnosis of religious, spiritual or mystical experiences from forms of psychopathology is an important but relatively neglected dimension of the psychology of religion. The investigation of how these criteria are applied in the context of contemporary communities of Western Buddhists suggests that determining the need for intervention is often a more important decision than appraising a given experience as either religious or psychopathological. Given both the heterogeneity of religious traditions and approaches within a tradition, and the fact that the current study found little consensus among Western teachers and practitioners of Buddhist traditions, it remains difficult to identify universal criteria for differential diagnosis. Although Buddhist traditions complicate the use of specific criteria such as distress, duration, and control, in particular, this does not render these criteria entirely irrelevant. In conjunction with other key determinants, such as a specific phenomenology, these criteria can be used to make practical appraisals and guide responses to meditation-related challenges. Although there were exceptions, functional impairment, loss of critical attitude, and certain phenomenological qualities—especially loss of ability to sleep, mania, delusions, suicidality and disruptive behaviors—were regularly used, especially by teachers, in determining need for intervention. Often these decisions took into account additional contextual factors such as the practitioner’s capacity to hold or respond skillfully to a challenge, as well as the clinical expertise or resources available in the community. This pragmatic approach is congruent with current approaches in cultural psychiatry that emphasize how experiences are inevitably appraised and navigated in relation to their social and cultural contexts (Kirmayer and Gómez-Carrillo, 2019). The Cultural Formulation Interview in DSM-5 and other methods of person-centered psychiatry provide ways to contextualize experience that can guide assessment and differential diagnosis (Kirmayer et al., 2016). Further work is needed to develop tools or frameworks for the assessment of meditation-related challenges based on the findings of this study.

## Data Availability Statement

The datasets presented in this article are not readily available because sensitive and potentially identifying information from this qualitative research, including original interviews, cannot be provided due to ethical restrictions. Requests to access the datasets should be directed to IRB@Brown.edu.

## Ethics Statement

The studies involving human participants were reviewed and approved by Brown University Institutional Review Board. The patients/participants provided their written informed consent to participate in this study.

## Author Contributions

JL assisted in project conceptualization, acquired project funding, conducted qualitative interviews, guided and contributed to the current data analysis, was the primary author of the first draft, was the primary reviser of subsequent drafts, and prepared the manuscript for submission. DC conducted qualitative interviews, assisted with the current data analysis, drafted subsections, and revised and edited first draft. NF conducted qualitative interviews, assisted with the current data analysis, drafted subsections, and revised and edited the first draft. LK contributed to subsections and revised and edited the first draft. WB led project conceptualization, acquired project funding, conducted qualitative interviews, contributed to subsections, and revised and edited the first draft. All authors contributed to the article and approved the submitted version.

## Conflict of Interest

The authors declare that the research was conducted in the absence of any commercial or financial relationships that could be construed as a potential conflict of interest.

## Footnotes

1 The ten spiritual emergencies identified were: (1) the shamanic crisis; (2) the awakening of *kuṇḍalinī*; (3) episodes of unitive consciousness (“peak experiences”); (4) psychological renewal through return to the center; (5) the crisis of psychic opening; (6) past-life experiences; (7) communications with spirit guides and “channeling”; (8) near-death experiences; (9) experiences of close encounters with UFOs; (10) possession states (Grof and Grof, 1989, pp. 13−14).

2 The present paper also includes data from a replication study of practitioners and experts in the Vipassanā tradition of S.N. Goenka. As a result, an additional eight practitioners and one expert were included this analysis.
